# Unveiling Hypereosinophilia's Stealthy Grip on Cerebral Sinus Venous Thrombosis: A Silent Association

**DOI:** 10.7759/cureus.60012

**Published:** 2024-05-09

**Authors:** Suprit Malali, Harshitha Reddy, Palash S Kotak, Sunil Kumar, Rushikesh H Dhondge

**Affiliations:** 1 Internal Medicine, Jawaharlal Nehru Medical College, Datta Meghe Institute of Higher Medical Research, Wardha, IND

**Keywords:** adult, stroke, corticosteroids, cerebral sinus venous thrombosis, hypereosinophilia

## Abstract

The report explores a case of cerebral sinus venous thrombosis associated with hypereosinophilia, presenting a unique clinical scenario. A 22-year-old male presented with persistent headache for eight days, escalating in intensity, along with projectile vomiting and blurred vision. Despite the absence of typical indicators such as fever or respiratory symptoms, comprehensive evaluations revealed hypereosinophilia in the complete blood count. Imaging studies, including magnetic resonance angiography and venography, confirmed cerebral sinus venous thrombosis. The patient was successfully treated with a multidimensional approach, including anticoagulation therapy, corticosteroids, and supportive measures. This report highlights the concealed nature of hypereosinophilia in the context of cerebral sinus venous thrombosis and underscores the importance of a vigilant diagnostic approach in unravelling this silent association.

## Introduction

Hypereosinophilia (HE) is a condition marked by a sustained elevation of eosinophilic count surpassing 1,500 cells/mm^3^. If end-organ damage is evident, then it is termed "hypereosinophilic syndrome (HES)," which often presents with a spectrum of clinical manifestations. These manifestations range from myocardial fibrosis to thrombotic complications.

Cerebral sinus venous thrombosis (CSVT) is a rare but potentially serious condition characterized by the formation of blood clots in the dural venous sinuses of the brain. These sinuses are responsible for draining deoxygenated blood from the brain back to the heart. CSVT can lead to impaired blood flow, increased intracranial pressure, and neurological deficits if not promptly diagnosed and managed. Typical causes of CSVT include hypercoagulable states such as pregnancy, oral contraceptive use, genetic thrombophilias, systemic infections, head trauma, and malignancies.

While the impact of HE on various organ systems is recognized, comprehensive documentation of cases linking HE to CSVT is notably lacking in the existing literature. This study aims to address this gap by presenting a distinctive case of idiopathic HES that predominantly affects cerebral sinuous venous structures, leading to the development of CSVT in a young adult. It endeavors to unravel the intricacies of this silent yet potentially devastating connection, providing insights into diagnostic considerations and therapeutic approaches.

## Case presentation

A 22-year-old male presented to the Emergency Department with a chief complaint of persistent headache for eight days, which increased in severity within the last two days prior to ED presentation. He reported experiencing four to five episodes of projectile vomiting and blurred vision within the last 12 hours. There was no history of fever, cough, cold, sore throat, or any active ear and nose discharge. The patient had no known comorbidities, allergies, medication intake, prior similar incidents, hospital admissions, or significant family history.

Upon a general examination, the patient was in an agitated state. His blood pressure was 130/90 mmHg, pulse rate was 88 beats/min, SpO_2_ was 98% on ambient air, and respiratory rate was 16 breaths/min. The cardiovascular assessment showed regular S1 and S2 heart sounds without any murmurs, S3, S4, or rubs. Respiratory system examination was normal, with no added sounds. An examination of the abdomen revealed a soft, non-tender abdomen without signs of organomegaly. On the central nervous system examination, the mental system examination was normal, cranial nerve examination was normal, and bilateral papilloedema was discovered during the fundus examination with grade 2 disc edema with unaffected visual acuity and visual field. Motor system examination was normal, deep tendon reflexes were present and normal, and bilateral plantar reflexes were flexor. Coordination, sensory system examination, and gait were normal.

Laboratory investigations such as complete blood count and metabolic panel were conducted, revealing HE in the complete blood count with an absolute eosinophil count (AEC) of 1,900 cells/mm^3^. Liver and renal function tests, coagulation profile, serum homocysteine levels, and serum IgE levels were within normal ranges (Table 1).

**Table 1:** Laboratory investigations of the patient.

Lab parameters	Observed value	Normal range
Hemoglobin	16 gm%	13–17 gm%
Mean corpuscular volume	89.2 fL	83–101 fL
Total leucocyte count	6,540 cells/cumm	4000–10.000 cells/cumm
Platelets	3.7 lakhs/cumm	1.5–4.1 lakhs/cumm
Serum urea	54 mg/dL	19–43 mg/dL
Serum creatinine	0.9 mg/dL	0.66–1.25 mg/dL
Serum sodium	132 mmol/L	137–145 mmol/L
Serum potassium	4.1 mmol/L	3.5–5.1 mmol/L
Serum calcium	9.2 mg/dL	8.4–10.2 mg/dL
Serum magnesium	2.0 mg/dL	1.6–2.3 mg/dL
Serum phosphorous	2.9 mg/dL	2.5–4.5 mg/dL
Serum uric acid	4.2 mg/dL	3.5–8.5 mg/dL
Alkaline phosphatase	130 U/L	38–126 U/L
Alanine transaminase	22 U/L	<50 U/L
Aspartate transaminase	48 U/L	17–59 U/L
Serum albumin	4.4 g/dL	3.5–5 g/dL
Total bilirubin	0.9 mg/dL	0.2–1.3 mg/dL
Conjugated bilirubin	0.3 mg/dL	0.0–0.3 mg/dL
Unconjugated bilirubin	0.6 mg/dL	0.0–1.1 mg/dL
Random blood sugar	134g/dL	90–140 g/dL
Serum vitamin B12	800 pg/mL	239–931 pg/mL
Serum vitamin D	24.27 ng/mL	20–40 ng/mL
Thyroid-stimulating hormone	0.522 mIU/mL	0.465–4.68 mIU/mL

Thrombophilia profile and tests for eosinophilia-associated diseases returned negative results (Table 2).

**Table 2:** Thrombophilia screening and eosinophilia-associated condition testing. CD, clusters of differentiation; Ig, immunoglobulin; PRIST, paper radioimmunosorbent test

Thrombophilia screening		
Protein S (%)	82	70–120
Protein C (%)	88	70–120
Antithrombin (%)	92	80–120
Activated protein C resistance	2.6	>1.5
Anticardiolipin IgG (U/GPL)	8	<20
Anticardiolipin IgM (U/MPL)	6	<20
Lupus anticoagulant	1.06	<1.3
Serum homocysteine	12 mmol/L	6.6–14.8 mmol/L
Leiden factor V gene variant	Wild type	Wild type
A20210G prothrombin gene variant	Wild type	Wild type
Eosinophilia-associated condition testing		
PRIST IgE (U/L)	240	0–260
C-reactive protein	76	10–1,000
Complement 3 (mg/dL)	95	80–180
Complement 4 (mg/dL)	18	15–50
Fecal pinworms	Absent	Absent
CD4 lymphocytes (%)	43	30–60
CD8 lymphocytes (%)	22	15–38

**Figure 1:**
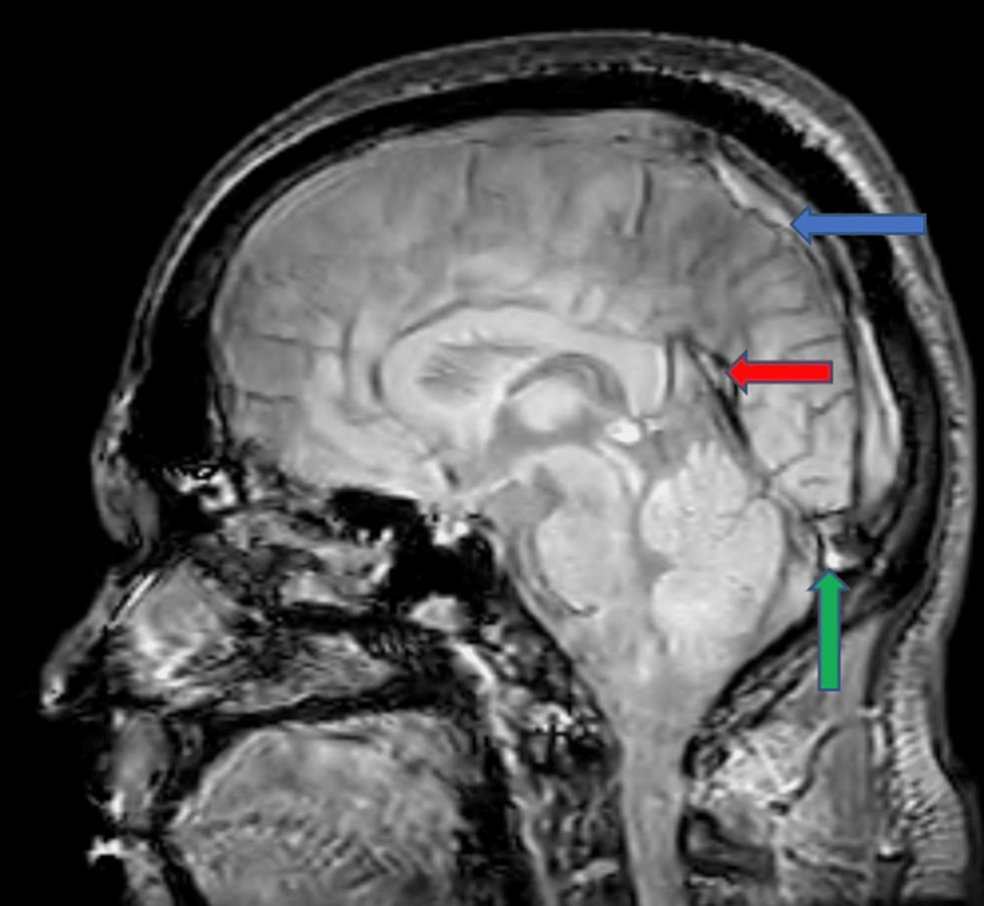
MRI of the brain showing loss of normal signal intensity in the superior sagittal sinus (blue arrow), straight sinus (red arrow), and transverse sinus (green arrow)

Stool examination and microscopy showed no abnormalities. Magnetic resonance imaging (MRI) of the brain showed loss of normal signal intensity in the superior sagittal sinus, straight sinus, and transverse sinus (Figure 1).

**Figure 2:**
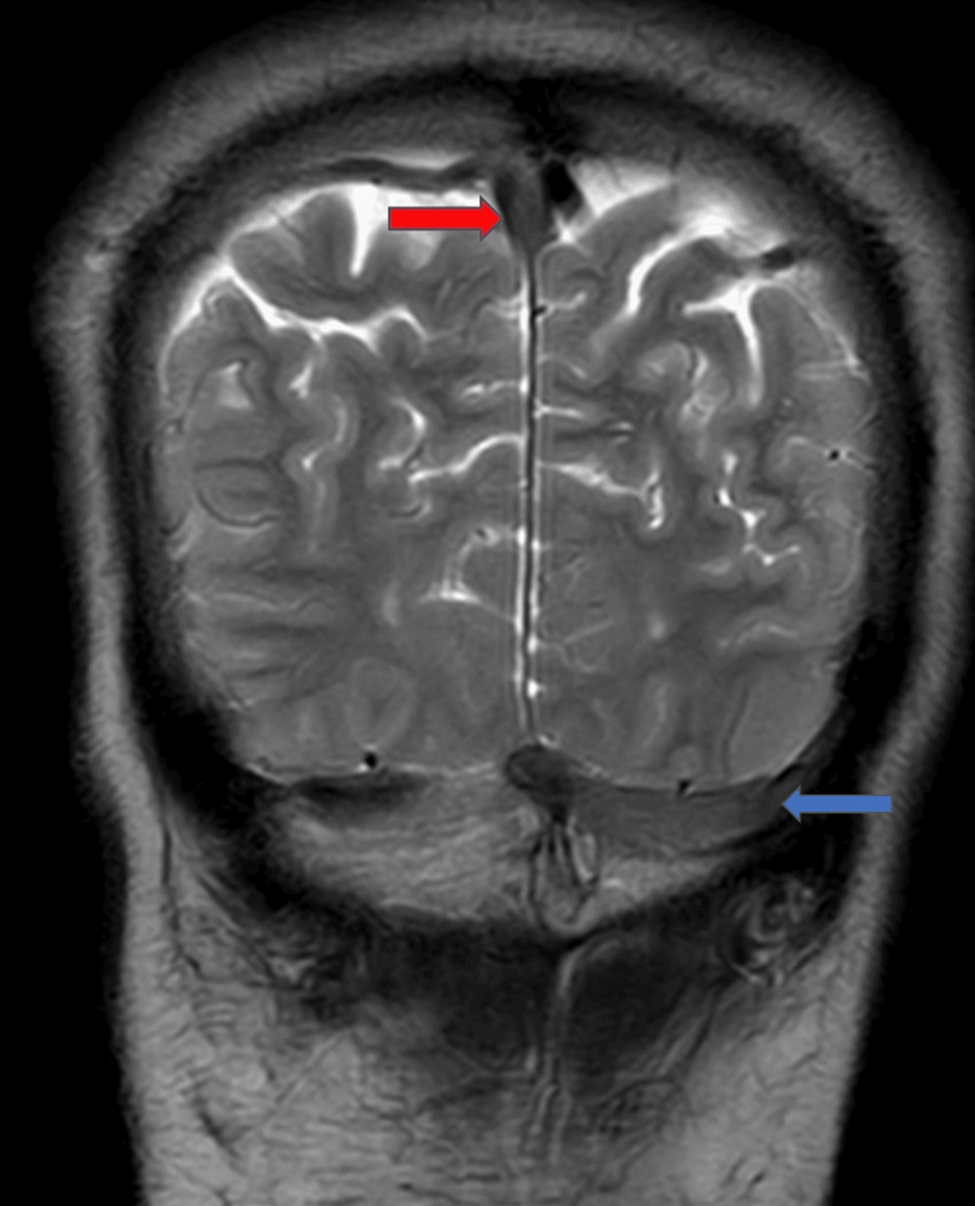
MRI of the brain showing loss of normal signal intensity in the superior sagittal sinus (red arrow) and transverse sinus (blue arrow)

MRI of the brain showed loss of normal signal intensity in the superior sagittal sinus and transverse sinus (Figure 2).

**Figure 3:**
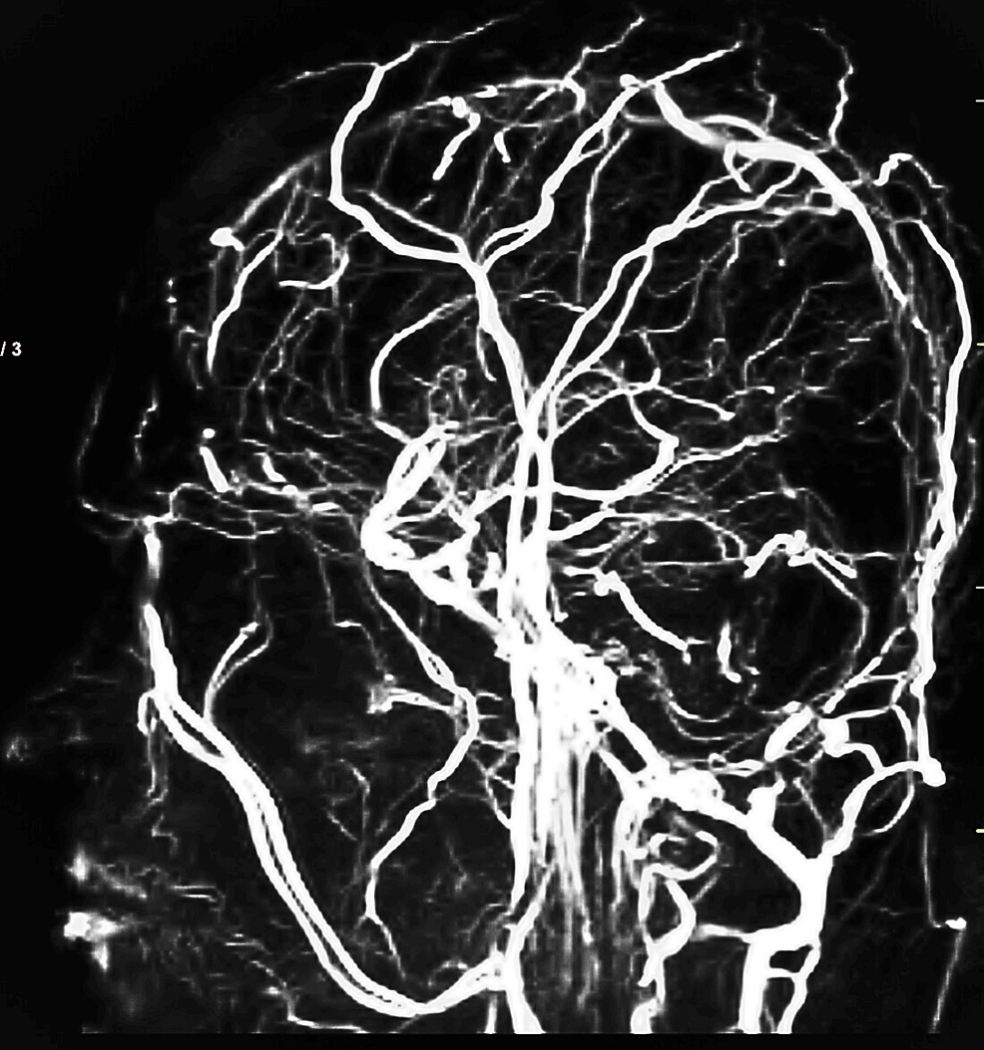
MRV of the brain showing loss of normal signal intensity in the superior sagittal sinus, inferior sagittal sinus, and transverse sinus MRV, magnetic resonance venography

Magnetic resonance venography of the brain showing loss of normal signal intensity in the superior sagittal sinus, inferior sagittal sinus, and transverse sinus (Figure 3).

The patient was initiated on treatment, including subcutaneous injection of low molecular weight heparin (0.6 mL twice a day), overlapped with oral tablet dabigatran (150 mg twice a day), intravenous injection of levetiracetam (1g twice a day), intravenous injection of mannitol (100 mL thrice a day), intravenous injection of tramadol (100 mg thrice a day), oral tab acetazolamide (250 mg thrice a day), and oral tab prednisolone (40 mg twice a day, tapered slowly every fifth day). The AEC began to decrease with the initiation of systemic steroids. The patient showed improvement in headache. On repeat fundus examination, there was a significant decrease in papilledema (grade 1), and the patient was discharged on the 12th day with an oral tab of dabigatran (150 mg once a day). The patient continues to attend regular follow-ups and is progressing well.

## Discussion

Eosinophils, constituting 3%-5% of circulating blood leukocytes, serve diverse functions in maintaining tissue homeostasis [1]. Eosinophilia encompasses both non-hematologic (reactive) and hematologic (clonal) disorders, potentially leading to end-organ damage [2]. Thrombosis represents a severe complication of HE, with approximately one-quarter of HES patients experiencing thromboembolic events, resulting in 5%-10% mortality [3]. While CSVT is rare, eosinophils contribute to a procoagulant state by releasing tissue factors and providing a phospholipid surface for thrombin generation [4]. Eosinophilic proteins activate platelets, and endothelial damage amplifies vascular permeability, contributing to a procoagulant environment. Thrombocytopenia may occur, potentially related to consumptive thrombocytopenia induced by thromboembolism [5].

Intracranial hemorrhage associated with HE has been observed in cases of CSVT, with potential mechanisms including secondary effects of CSVT, thrombocytopenia, anticoagulant drug side effects, or direct endothelial injury from eosinophilic infiltration [6]. However, in our case, there was no evidence of intracranial bleeding. Eosinophilia lacks a uniform standard for classification, but HES can be categorized into hereditary, primary, secondary, or idiopathic types. CSVT can be induced by HE, emphasizing the importance of monitoring eosinophil counts in CSVT patients [7]. Commencing steroid therapy promptly could mitigate disease advancement, given the association between sustained eosinophilia and an elevated likelihood of thromboembolism recurrence [8]. The incidence of venous thrombosis (VT) in patients with HE is recognized as a defining characteristic of HES per the most recent criteria for categorizing eosinophilic conditions and related syndromes developed for eosinophil disorders by the International Cooperative Working Group (ICOG-EO) [9].

Only five cases of VT in eosinophil-related disorders have been reported in the biggest interdisciplinary worldwide collaborative series, which included 188 HES patients in all subtypes of diseases, despite this classification. The European Society of Cardiology's guidelines do not specifically list HE as a potential factor for the development of venous thromboembolism [10]. This discovery is noteworthy. Presently, there are limited clear guidelines for treating VT that arises in the presence of HES. The shortage of documented cases and the lack of specific guidelines underscore a substantial gap in our comprehension of the association between HE and thrombosis. Further research and clinical investigations are warranted to elucidate the mechanisms and risk factors associated with VT in the context of eosinophil-related disorders. Until comprehensive guidelines are established, individualized and thoughtful management strategies should be employed in the clinical care of patients presenting with both HE and VT.

## Conclusions

HES can affect multiple organs, necessitating comprehensive investigations to rule out other causes of HE. The link between the condition and hypercoagulability emphasizes the critical need for vigilance, particularly in efforts to reduce a significant mortality rate. Early diagnosis remains challenging, emphasizing the need for prompt initiation of corticosteroids, particularly in severe or life-threatening conditions such as CSVT. In cases resistant to corticosteroids, alternative therapies may be necessary. Managing thromboembolic diseases associated with HES poses particular challenges.
